# Nitric oxide for cancer therapy

**DOI:** 10.4155/fso.15.44

**Published:** 2015-08-01

**Authors:** Sergio Huerta

**Affiliations:** 1 Department of Surgery, UT Southwestern Medical Center, VA North Texas Health Care System, 4500 S. Lancaster Rd./Surgical Services (112)/Dallas, TX 75216, USA

**Keywords:** diazeniumdiolates, JSK, NO donors, NO hybrids

## Abstract

Resistance to current therapeutic interventions is a major challenge in the treatment of patients affected by cancer. While nitric oxide (NO) might have proneoplastic properties, it is now clear that at high doses, NO has a role in cancer therapeutics. Either as a single agent or in combination with other antineoplastic compounds, NO might be used to overcome tumor cell resistance to conventional treatments. The following discussion addresses the role of NO in cancer therapeutics and includes a report on the role of NO donors in the area of cancer therapeutics.

**Figure 1.**
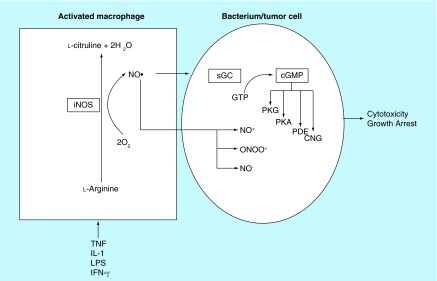
**Activated macrophages liberation of nitric oxide resulted in cytostatic and cytotoxic activity of target cells.** These were the initial observation that led to the interest of nitric oxide in cancer biology. cGMP: Cyclic GMP; CNG: Cyclic nucleotide-gated channels; LPS: Lipopolysaccharide; NO: Nitric oxide; PDE: Phosphodiesterase; PKA: Protein kinase A; PKG: Protein kinase G; sGC: Soluble guanylyl cyclase. Adapted from [1].

**Figure 2.**
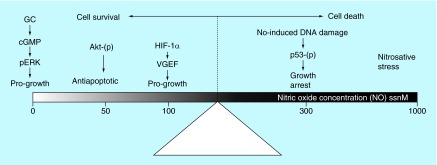
**The concentration of nitric oxide determines its role in tumorogenesis.** At low concentration, NO causes tumor progression. At high concentrations of NO, anticancer activity is observed. GC: Guanylyl cyclase; GMP: Guanosine monophosphate; HIF: Hypoxia inducing factor; NO: Nitric oxide. Adapted from [5].

**Figure 3.**
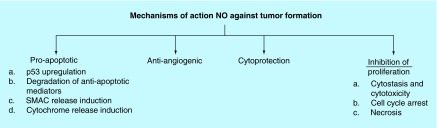
**Mechanisms of action that have been investigated leading to the anticancer properties of nitric oxide**. NO: Nitric oxide.

**Figure 4.**
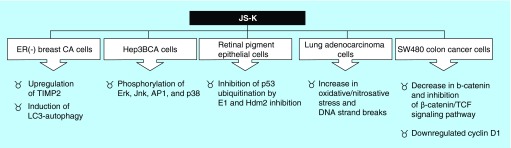
**JS-K has a wide array of antitumor properties.**

Nitric oxide (NO) is a ubiquitous, water-soluble, free radical gas that exerts a wide range of biological effects. NO is generated by the oxidation of the amino acid l-arginine under the catalytic activity of the NO synthases (NOSs), which requires NADPH and O_2_ as cosubstrates to yield NO and l-citrulline as end products. The wide array of NO mediated functions occur largely via a cGMP-dependent pathway, which lead to vasodilation, neurotransmission, inhibition of platelet aggregation and smooth muscle relaxation. A second pathway is cGMP-independent and occurs by the reaction of NO with molecular O_2_, superoxide (O_2_^-^) thiols and transition metals such as zinc [1]. NO can also modify proteins directly without the use of enzymes such as by nitration or nitrosylation. S-nitrosylation of cysteine thiol residues is a reversible modification involved in cell signaling, which regulates the function of many intracellular proteins [1].

NO is a small molecule, but received a great deal of attention in 1992 by *Science* as the molecule of the year, led to a Nobel Prize Award in 1998, and has its own journal and society. It has been the role of NO in the cardiovascular system that has led to this magnificent impetus. However, a new interest has emerged over the past couple of decades that implicates NO in carcinogenesis and tumor growth inhibition. The actions of NO in cancer are variable with respect to its role as an antineoplastic versus a proneoplastic agent. This variability stems from the dose of NO. The role NO in cancer therapeutics has become even more notable as a result of multiple NO donors that have been introduced over the past few decades and the recent development of novel hybrid drugs.

In 2007, Drs. Bonavida and Jeannin organized the First International Conference of Nitric Oxide and Cancer (NO Cancer), which brought international leaders on the field of NO. This is a testament of the importance of NO in cancer therapeutics and continues to generate more and more interests as more drugs are being introduced. The present review focuses on the role of NO as an antineoplastic agent either as a cytotoxic agent by itself or as a sensitizer to overcome chemo radioresistance to conventional treatments

## Antineoplastic properties of NO

In 1985, reports emerged that documented the production of nitrite (NO_2_^-^) and nitrate (NO_3_^-^) *in vitro* by macrophages when induced by lipopolysaccharide and IFN-γ [2]. NO_2_^-^/NO_3^-^_ synthesis was dependent of l-arginine and led to cytotoxicity of bacteria and tumor cells (Figure 1) [2,3]. These initial observations initiated the concept that led to a potential role of NO as an anticancer agent. Reports of the role of NO in cancer rapidly accumulated, but divided its role by virtue of its biphasic activities as an antineoplastic agent and a proneoplastic molecule [4]. At low levels, NO can lead to tumor formation. The mechanisms of action that lead to the pro-neoplastic activity of NO are numerous, but include: cell proliferation by activation of oncogenes; stimulation of angiogenesis; apoptosis inhibition by: S-nitrosylation-inactivation of caspases- 1, 2, 4, 8 and 3, 6, 7, inhibition of apoptosis by disruption of the Apaf-1/Caspase-9 complex (32), induction of heat shock protein 70 (Hsp 70), mutation of *p53* (33–35) and activation of COX-2. Certainly, a number of processes can occur simultaneously leading to this multifactorial effect.

The role of NO as an anti-oncogenic agent has also been well established to comparable degree as its potent anticancer properties in other reports. Thus the dual role of NO is well established and documented in the medical literature. Thus, it continues to be of crucial importance to investigate the system under study to evaluate the contribution of NO to the environment in which it is being released.

In 2008, David Wink's group put much of this controversy to rest by reporting a specific concentration threshold where the bipartisan role of NO occurred (Figure 2) [5]. At high concentrations (>200 nM), NO had an anticancer properties; whereas below this threshold, cell survival and a pro-neoplastic function of NO was observed. These observations provided a clear delineation of such biphasic role of NO in cancer. However, in a particular system *in vivo* or in human trials, it remains difficult to establish the concentration of NO, the half-life available to lead to the anticancer properties, the multiple reactions with other molecules and other reactive properties of NO.

Recognizing that, *in vitro* at low concentration, NO has proneoplastic properties is important in any investigation of NO in cancer biology. These effects might be mediated by: facilitating cancer cell proliferation [6]; inhibition of apoptosis [7]; stimulation of angiogenesis [8]; and promoting genomic instability [9]. S-nitrosylation and nitrozation play a key role in the proneoplastic properties of NO [7,9]. Once the specific pathways leading to carcinogenesis have been elucidated, the role of NO at high concentrations can be further explored.

It is now evident that at high concentrations NO has a potential role in cancer therapeutics. Such role must take advantage of the tumor cell microenvironment, the tumor cell background in terms of mutations, the amount and duration of exposure of NO to the targets cells. *In vitro*, cytostatic effects were observed via NO derived from macrophages, Kupffer cells, Natural Killer cells and well as endothelial cells [10]. *In vivo* models that over express inducible NO synthase (iNOS) have demonstrated an antineoplastic properties of NO [1]. Some of the proposed mechanisms of action that account for the antitumor properties of NO are depicted in Figure 3 [1].

NO has a short half-life and rapidly reacts with other compounds. For instance, NO produced from neuronal NOS (nNOS) and endothelial NOS (eNOS) has cellular effects on the order of seconds to hours and generate NO in the nanomolar range. Inducible NOS produces NO at concentrations between 0.1 and 4.0 μM over a temporal window that can convert hours to days. Of the several biological activities of iNOS, its role in the immunogenic and cytotoxic T-lymphocyte responses as well as its bacteriostatic activity on reticuloendothelial cells have rapidly evolved in the study of NO in oncology. However, even with larger concentrations and wider range of availability, the NO produced from intrinsic sources might have an unpredictable response to multiple cancer cells and tumors. Thus, the low doses and the short half-life of NO delivered from intrinsic sauces, limit their study in cancer therapeutics.

The introduction of NO donors and their further development and refinement have allowed for a more controllable and predictable application of NO in cancer biology. The large variety of NO donors allows for selection of a compound that can release NO with predictable concentrations and with a wide range of time release. Thus, NO donors have permitted a more robust analysis of the role on NO in cancer therapeutics.

## NO donors

As new compounds are constantly introduced, it has become more evident that there is a tremendous role of NO donors in cancer treatment. However, the use NO donors is not without limitations. For instance, the byproduct of an NO donor can be toxic to the cell (i.e., sodium nitroprusside [SNP], which generates cyanide as its byproduct). NO itself has potent vasodilatory effects which can have a profound undesirable effects on the cardiovascular system. The microenvironment of a cell might lead to reactions difficult to control such as pH and temperature, which might alter the release of NO. The specific enzymatic reaction required for the release of NO might differ widely within cancer cells.

However, there are innumerable benefits in using NO donors in cancer. NO donors have a wide range of time release (i.e., 1.8 s to 56 h depending of the NO donor). Multiple mechanisms of action as a result of the NO parent compound can be elicited. A more promising role of NO donors is with its potential in targeted delivery to tumor cells. For instance, several NO prodrugs are designed in such a way that they undergo activation by enzymes particularly over expressed in cancer tissue (i.e., glutathione *S*-transferase).

There are multiple schemes to classify NO donors [1]. A common classification is based on the structure of the NO donor and the form in which NO is generated (i.e., enzymatic vs nonenzymatic). In this discussion, a limited classification is presented based on the role of NO-donors in cancer treatment.

## NO donors in cancer

Because of the potential systemic effects exerted *in vivo* by NO donors such as hypotension and accumulation of toxic metabolites, an ideal NO donor must be one that minimizes systemic adverse effects while maximizing its anticancer properties. Such is the rationale of the development of the NO hybrids, where an NO moiety is attached to a second antiproliferative agent (i.e., the COX-2 inhibitor [aspirin]). This provides an opportunity to explore the synergistic properties of both compounds while minimizing the adverse systemic effects of the high doses of each individual compound. Current trials are under way (i.e., NO-aspirin in high-risk patients with colorectal cancers [11]).

A large number of NO donors have been developed for the study of NO in tumor biology [1]. The following classification has been structured based on the current utilization of NO donors in cancer therapeutics [11]. Of these donors, the group belonging to the class of the *N*-Nitroso compounds (diazeniumdiolates [NONOates]) continues to gain interest in the area of oncology as more evidence emerges in their role as antineoplastic agents. There is also a new array of novel compounds belonging to this class that are being investigated as a result of their high antiproliferative properties.

In the present discussion is focused on NO donors being examined in cancer research and the results in several studies outlining anticancer properties and the possible role of NO donors in cancer therapeutics for the management of human malignancies. The discussion of NO donors follows the following general outline.

### Organic nitrates

Organic nitrates include compounds such as: glyceryltrinitrate (GTN) and isosorbidedinitrate (ISDN). These are the oldest class of NO donors. Several studies have demonstrated anticancer properties of these compounds *in vitro* and *in vivo. In vitro*, GTN inhibited the metastatic potential of murine melanoma cells and led to apoptosis sensitization in colon cancer and prostate cancer cells to doxorubicin. *In vivo*, GTN prevented the formation of murine melanoma cell lung nodules [1].

GTN transdermal patches chemosensitized prostate cancer xenografts to doxorubicin [12]. Nitroglycerine patches have been shown to have a therapeutic effect on small cell lung cancer patients when added to standard chemotherapeutic drugs. These effects occurred with minimal side effects from the use of the transdermal patches [13].

### Metal-NO complexes (sodium nitroprusside)

These compounds are generated as a result of the great affinity of NO to metals. The most common compound used as an anticancer agent is SNP (Na_2_Fe(CN)_5_NO). SNP has been shown to have anticancer properties in prostate, gastric and cervical cancer cells as well as radiosensitization in pancreatic and glioma cancer cells. Similarly, an increase in apoptosis was observed in T-cell lymphoma cells treated with SNP [1].

### S-nitrosothiols

The two compounds typically used as antineoplastic agents are: S-nitroso-*N*-acetylpenicillamine (SNAP) and S-nitrosoglutathione (GSNO). These compounds have the general formula ‘RSNO’, and, as a class, are typically unstable. However, SNAP is relatively stable and functions as an NO donor with a potent vasodilator activity. SNAP has been shown to have anti-apoptotic properties *in vitro*. In addition, SNAP has demonstrated substantial radiosensitization in several cancer cell lines such as cervical (HeLa), Glioma, Chinese hamster V79 lung cells and murine mammary adenocarcinoma EMT-6 cells. GSNO also has relative stability and serves as a source of NO based on the cleavage of the S-NO bond. GSNO induced apoptosis and cell cycle arrest in colon cancer cells and radiosensitized hypoxic Chinese hamster V79 lung cells [1].

### Sydnonimines

A typical compound that has been studied in this class is 3-morpholinosydnonimine (SIN-1). SIN-1 generates peroxynitrite (OONO-), which induces cellular damage by causing single stranded DNA breaks, induces protein nitration and inhibits mitochondrial respiration [1]. Peroxynitrite from SIN-1 caused neurotoxicity in rodent cortical cells. SIN-1 had antineoplastic properties against glioma C-6 cells and induced neuronal cell death as well and apoptosis induction in lymphoblastoid WTK cells [1]. In esophageal cancer cells, SIN-1 in combination with the carcinogen myosimine led to an increase in nitrosative stress [14].

### Diazeniumdiolates (NONOates)

These compounds are characterized by the basic structure: X- [N(O)NO]-, in which ‘X’ is typically a secondary amine. In general, this group of compound is known as ‘NONOates’ and have been extensively studied in cancer therapeutics *in vitro* and *in vivo* as a result of the wide range of compounds with a large spectrum of half-lives (2 s to 20 h). Keffer's group at the NCI have generated a large number of these compounds [15]. The main compounds in this group are depicted in Table 1. Their accepted nick names as ‘NONOates’ rather than the chemical formal name are classically utilized. The compounds in this group include: DEA/NO, PAPA/NO, SPER/NO, PROLI/NO, MAHMA/NO and DETA/NO and JS-K. The major properties of these compounds in cancer therapeutics are discussed below.

## DEA/NO & PAPA/NO

The properties of DEA/NO (Table 1) alone and in combination with other NO-donors have been studied a potential agents in cancer therapeutics *in vitro* and *in vivo*. DEA/NO demonstrated antiproliferative properties against breast cancer cells and decreased their metastatic potential to bone. DEA/NO also had antiproliferative properties on NB69 neuroblastoma cells [1]. Evidence of the role of PAPA/NO (Table 1) as a potential antineoplastic agent was originally suggested by radiosensitization of hypoxic murine mammary adenocarcinoma cells by IFN-γ-induced iNOS upregulation resulting in the increased levels of NO and apoptosis induction in HT29 colon cancer cells [1].

## SPER/NO, PROLI/NO & MAMA/NO

SPER/NO (Table 1) had radiosensitizing properties in hypoxic murine mammary SCK cells to a similar magnitude as DEA/NO. At high doses of DEA/NO (100 to 500 µM), cell cycle arrest and decreased proliferation was observed in salivary gland (HSG) cells. Low doses of the same compound led to opposite effects. PROLI/NO inhibited smooth muscle in canine endarterectomized arteries *in vivo*. An increase in survival was observed in rats with C-6 gliomas receiving combination treatment with carboplatin and PROLI/NO compared with carboplatin, PROLI/NO or vehicle alone [1]. MAHMA/NO has a limited role in as an antiproliferative agent in compared with other NO donors. In HT29 colon cancer cells, MAHMA/NO was not as effective in suppressing essential enzymes for proliferation (Ornithine decarboxylase [ODC]) as SNP [1].

## DETA/NO

The sustained release of NO (over 20 h) from DETA/NO (Table 1) make it an attractive compound to be used in cancer therapeutics. DETA/NO induced cytostasis and cell cycle arrest in human breast cancer cells MDA-MB-231 as well as apoptotic cell death. In spheroid cultures of breast cancer cells, DETA/NO and GTN attenuated the doxorubicin resistance. DETA/NO, in low doses, reversed the hypoxia-mediated resistance to chemotherapeutic agents such as 5-FU and doxorubicin in human breast carcinoma MDA-MB-231 cells and B16F10 mouse melanoma cells [1]. DETA/NO enhanced cisplatin mediated toxicity in Chinese hamster V79 lung fibroblasts and head and neck squamous cell carcinoma cells. In melanoma xenografts, mice receiving DETA/NO in addition to cisplatin had a significant reduction of tumors an increased survival compared with mice treated with cisplatin or vehicle alone. DETA/NO immunosensitized prostate cancer PC-3 cells to TRAIL- and FasL-mediated apoptosis. DETA/NO chemosensitized SW620 xenografts to cisplatin mediated AIF-apoptosis [16] and led to profound radiosensitization of highly radioresistant HT29 cells and xenografts [17].

## JS-K

Of several NO donors designed by Joseph Saavedra, one contained in the ‘K’ tube demonstrated the highest antitumor activity. This compound became to be known as JS-K (personal communication: L. Keffer, 2012). The compound JS-K is the prodrug: *O^2^*-(2,4-dinitrophenyl) 1- [(4-ethoxycarbonyl)piperazin-1-yl]diazen-1-ium-1,2-diolate, that has shown antitumor activity in a variety of rodent cancer models (Table 1). JS-K was designed to be activated by reaction with glutathione (GSH) to release two moles of NO. The anticancer properties of JS-K are centered on two principles. The first one is based on the fact that cancer cells often overexpress glutathione *S*-transferase, which allows drug specific enzymatic action in tumor tissue, while sparing potential systemic side effects. For instance, JS-K was a potent anticancer agent against HL-60 leukemia cells and caused a substantial reduction of implanted xenografts without hypotensive events. The second principle has to do with the anticancer activity of NO.

JS-K has been shown to have potent antineoplastic activity against many tumor types *in vitro*. Additionally, *in vivo* JS-K has proved activity against different cancers such as leukemia xenografts in mice, murine prostate cancer xenografts, orthotopic models of liver cancer in rats, non-small-cell lung xenografts in mice, gliomaxenografts in rats and multiple myeloma xenografts in mice. Furthermore, JS-K has shown synergism in combination with other chemotherapeutic modalities including: bortezomib, sodium arsenite, cytarabine and cisplatin (Figure 4) [18].

### NO-drug hybrids

The systemic effects of NO in vasodilatation have led to the development of hybrid drugs where each compound can act in synergism to provide antitumor effects. This rationale ignited the development of NO linked to a COX-2 inhibitor (NO-NSAIDs: Table 2). The NO-aspirin NCX-4016 demonstrated no toxicity when provided to human subjects while maintaining COX-1 and antiplatelet activity. NO-NSAIDs are composed of typical nonsteroidal anti-inflammatory drugs such as aspirin, salicylic acid, indomethacin, ibuprofen or sulindac to which an NO-releasing moiety has been attached via a covalent bond that is cleaved by nonspecific esterase activity [11].

A wide array of NO-NSAIDs are currently being investigated for the role in cancer therapeutics. The compounds designed by Thatcher's group [19] are designated NCX-# (i.e., NCX-4016, NCX-4040, NCX-4215, NCX-976, and so on), several of which have been studied in oncology and a substantial number of compounds are on current randomized trials. NCX-4016 & NCX-4215 (NO-Aspirins) are compounds that have an aspirin moiety attached to NO. These compounds have demonstrated substantial anticancer properties *in vitro* and *in vivo* even in COX-2 negative cells [20].

A different groups of NO donors bound to a statin compound (i.e., pravastatin [NCX-6550]) inhibited cell proliferation in rat aortic smooth muscle cells this effect was accompanied by suppression of iNOS and COX-2 [21]. All of these compounds demonstrate a great deal of promise in the role of NO in cancer therapeutics.

**Table 1.** **Structure and half-life of diazeniumdiolates.**

**Name**	**Half-life**	**Structure**
DEA/NO	2.0 min	
PAPA/NO	15 min	
PER/NO	10–90 min	
PROLI/NO	2 s	
MAHMA/NO	1 min	
DETA/NO	20 h	
JS-K	26 min	

**Table 2.** **Nitric oxide drug-hybrids.**

**Name**	**Structure**
NO-Aspirin	
NO-indomethacin	
NO-Ibuprofen	
NO-Salicylic Acid	
NO-Sulindac	

NO: Nitric oxide

**Executive summary**

The literature demonstrated a dichotomy regarding the role of nitric oxide (NO) in cancer biology. However, it is now well established that at high concentrations NO has antiproliferative properties and has a great deal of potential in cancer therapeutics.
The drastic emergency of novel NO donors and NO hybrids is a testament of the activity and advancement of this field.
Substantial evidence *in vitro* and *in vivo* has accumulated to demonstrate the role of NO as an anticancer agent. However, clinical trials are still limited.
The diazeniumdiolates (NONOates) are promising NO donors as they have been shown to be effective chemo-and-radiosensitizing agents.
Some older NO donors such as glyceryltrinitrate (from transdermal patches) have been used for several years and their clinical activity demonstrated to be safe. Additionally, clinical trials have demonstrated efficacy for the management of resistant or metastatic tumors.
NO-hybrids appear to be making their way to clinical trials, but the verdict on these is still pending. Either as a single agent or as a chemo-radio-sensitizing additive,
NO continues to demonstrate more contributions in cancer therapeutics.
